# CRISPR-Cas immunity, DNA repair and genome stability

**DOI:** 10.1042/BSR20180457

**Published:** 2018-09-21

**Authors:** Andrew Cubbon, Ivana Ivancic-Bace, Edward L. Bolt

**Affiliations:** 1 School of Life Sciences, University of Nottingham, QMC Medical School, Nottingham, U.K.; 2 Department of Molecular Biology, Faculty of Science, University of Zagreb, Croatia

**Keywords:** CRISPR-Cas, DNA repair, HelQ

## Abstract

Co-opting of CRISPR-Cas ‘Interference’ reactions for editing the genomes of eukaryotic and prokaryotic cells has highlighted crucial support roles for DNA repair systems that strive to maintain genome stability. As front-runners in genome editing that targets DNA, the class 2 CRISPR-Cas enzymes Cas9 and Cas12a rely on repair of DNA double-strand breaks (DDSBs) by host DNA repair enzymes, using mechanisms that vary in how well they are understood. Data are emerging about the identities of DNA repair enzymes that support genome editing in human cells. At the same time, it is becoming apparent that CRISPR-Cas systems functioning in their native environment, bacteria or archaea, also need DNA repair enzymes. In this short review, we survey how DNA repair and CRISPR-Cas systems are intertwined. We consider how understanding DNA repair and CRISPR-Cas interference reactions in nature might help improve the efficacy of genome editing procedures that utilise homologous or analogous systems in human and other cells.

## Interplay of DNA repair and CRISPR-Cas immunity: the fundamentals

### Overview

CRISPR-Cas is a naturally occurring adaptive immunity system in prokaryotes [1,2]. Operational efficiency of CRISPR-Cas enzymes is closely associated with active DNA repair and replication, in natural CRISPR-Cas systems to promote building of adaptive immunity, processes called ‘Adaptation’, and in biotechnology where genome-editing reactions that utilise ‘Interference’ reactions also trigger DNA repair and their associated reactions. Identities of DNA repair enzymes involved in supporting native CRISPR-Cas systems in bacteria are becoming clearer, but the molecular mechanisms are not known or are inferred from known DNA repair and genome stability functions. Understanding these mechanisms might aid development of strategies for interpolating CRISPR-Cas enzymes (e.g. Cas9 and Cas12a, the latter formerly known as Cpf1) into eukaryotic cells, including in humans. This rationale is based on conservation of fundamental principles, and some specific properties, of DNA repair in bacterial and eukaryotic cells. New information is also emerging on how DNA repair processes in human cells support genome editing, which deepens understanding of how DNA repair systems are triggered and function in human cells, which can help to protect against cancers and other aging syndromes.

## CRISPR-Cas adaptive immunity and Cas9-based editing

**Figure 1:**
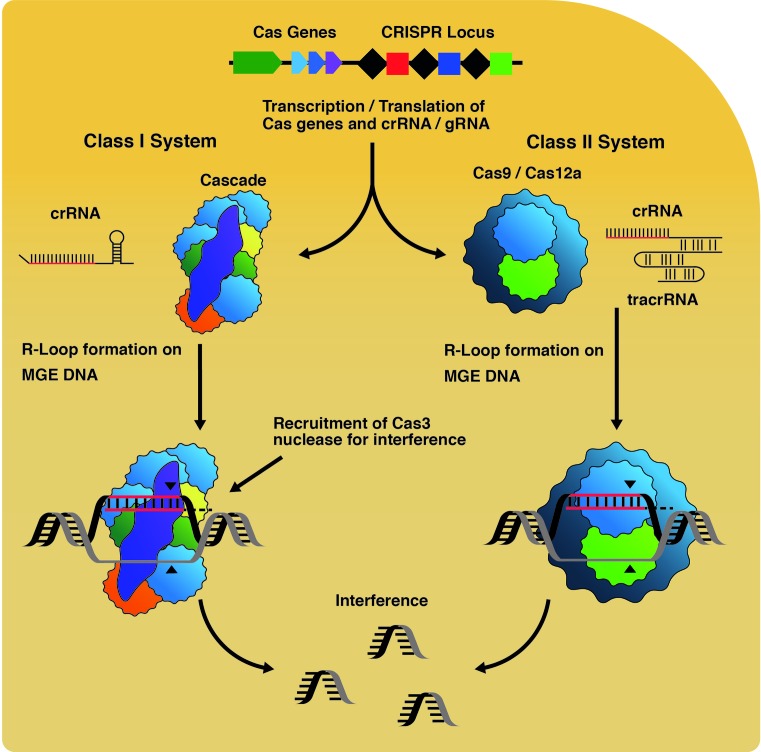
CRISPR-mediated interference reactions DNA from MGEs provide small ‘protospacer’ fragments for acquisition into a CRISPR-locus during ‘Adaptation’ that generates immunity. Transcription and processing of CRISPRs create crRNAs, which are loaded into Interference complexes. Cas9 requires a second, long trans-activating crRNA (tracrRNA), which base pairs with crRNA to produce a mature sequence. These complexes catalyse R-loop formation on target DNA leading to nuclease activity targeted to the R-loop site, catalysed by Cas3 recruited to Cascade, or by Cas9.

Deliverance of CRISPR-Cas immunity in native systems is through ‘Interference’ reactions that feature nucleotide base pairing of CRISPR-encoded RNA (crRNA) with an ‘invader’ mobile genetic element (MGE, e.g. a phage, plasmid). This is catalysed by a ribonucleoprotein Interference complex, also called an effector complex (Figure 1), reviewed in [3]. The molecular events within interference complexes vary according to the class and subtype of CRISPR-Cas system [4], but they incapacitate the MGE by binding to it stably, and triggering nucleolytic degradation of the MGE. Multi-subunit Cascade interference complexes of class I CRISPR systems [5], lack intrinsic nuclease activity but recruit the Cas3 nuclease-translocase enzyme to complete interference reactions [6,7]. Unlike Cascade, class 2 interference complexes have intrinsic DNA cutting activities. Co-incident DNA nicks generated by two nuclease active sites in Cas9 interference complex (RuvC-like and HNH-like) generates a DNA double-strand break (DDSB) [8,9]. Cas12a also generates DDSBs via two nuclease active sites and possesses potent ssDNA endo/exonuclease activity that has spawned further useful applications [10]. Structure, function and detailed mechanism of Cas9 and Cas12a are presented in a recent review [11].

Understanding molecular details of RNA-DNA base pairing in Cas9 interference reactions allowed for engineering of programmable single-guide RNAs to target DNA sequences of choice (sgRNA, Figure 1) [8], opening up the simplified DNA editing process that is now widely used for targeting individual genes. The effectiveness of using Cas9 for gene editing in cells is highlighted in landmark papers describing the first methods for editing genes in bacteria [12] and in human and mouse cells [13–15].

In native CRISPR-Cas systems, including the class 2 systems utilising Cas9, the crRNA payload that base pairs to DNA during interference is derived from transcription and processing of a CRISPR locus, in which each crRNA sequence is stored as a DNA ‘spacer’ (Figure 1). By engineering a CRISPR locus with multiple desired spacer sequences and transplanting into cells the engineered CRISPR with Cas9, and associated Cas proteins from the native system, it was possible to ‘multiplex’ Cas9 for targeting multiple genes as part of the same process [14].

RNA-DNA pairing by Cas9 forms the basis for genome editing, exploiting the molecular biology of native Interference reactions that target MGE DNA in R-loop nucleoprotein complexes [16,17] (Figure 1). The details of interference R-loop formation have been assessed in detail elsewhere [18,19]. There is currently a great deal of interest in how specificity for targeting of precise DNA sequences is achieved by Cas9 et al., and in off-site or genome instability effects of editing processes [20]. Both DDSBs and R-loops generated by Cas9, and other editing enzymes, have the potential to provoke genome instability by disrupting polymerases and helicases of DNA replication and transcription. Therefore CRISPR-Cas interference may trigger genome instability and cell death analogously to naturally occurring endogenous and exogenous genotoxins [20,21]. These lesions and blocks are detected globally or when linked to replication and transcription and dealt with by DNA repair systems that also impact on CRISPR-Cas interference reactions.

## DNA repair at DNA breaks

**Figure 2:**
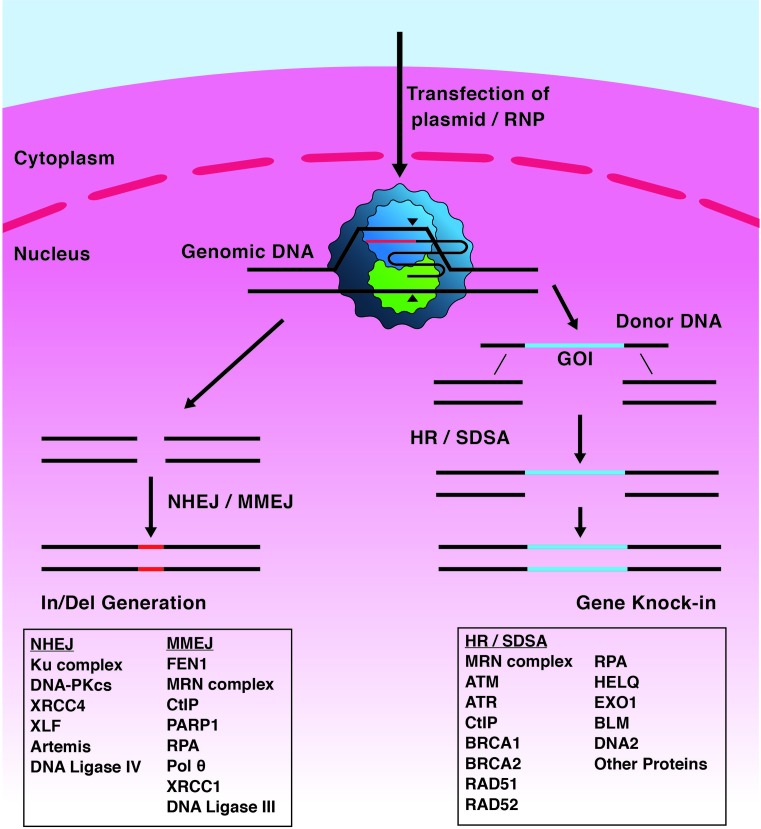
Overview of CRISPR-Cas9 triggered DNA repair Cas9 targets and cuts DNA at R-loops. Editing procedures arising from the resulting DDSB depend on host DNA repair systems; NHEJ or microhomology-mediated end joining (MMEJ) pathways can lead to the generation of insertions or deletions in the DNA, generating frameshifts, preventing gene expression. Homologous recombination (HR) or synthesis-dependent strand annealing (SDSA) can be utilised for the precise knockin of genetic material, for example a ‘gene of interest’ (GOI). All of these processes require the co-ordination of a large suite of proteins, the interactions of which with Cas proteins is poorly documented.

DNA repair systems most relevant to this summary are illustrated in Figure 2. Repair of DDSBs by **Non-Homologous End Joining** (NHEJ) proteins does not require DNA sequence homology but instead ligates broken DNA ends together. NHEJ is not present in many bacterial clades, but is characterised in *Bacillus subtilis, Pseudomonas aeruginosa* and *Mycobacteria* [22,23]. The genetic requirements and biochemical mechanisms for NHEJ in bacteria differ from eukaryotes. In eukaryotes NHEJ predominantly occurs in G_1_ phase of the cell cycle, reviewed in [24], and is inhibited during mitosis to prevent undesirable chromosome fusions at telomeres [25]. NHEJ is promoted by the Shieldin complex [26–29] and initiates from the Ku protein complex accessing exposed DNA ends. This serves as a scaffold for the recruitment of an assortment of lesion-specific accessory proteins including DNA-PKcs that stabilises broken DNA ends [30]. Artemis nuclease complex is recruited for DNA end processing, and DNA Ligase IV seals processed DNA ends to fix the break [31]. NHEJ is associated with insertion/deletion (In/Del) mutations several base-pairs long, which can induce frameshift in the coding regions of proteins, leading to their truncation and inactivity following translation [32].

DNA repair by **microhomology-mediated end joining** (MMEJ), also called Alternative End-Joining (A-EJ) [33], is also error-prone, and like NHEJ results in In/Del mutations, but MMEJ relies on 5–25 bp of sequence complementarity (‘microhomology’) between DNA strands by mechanisms that are still being worked out. A complex of Mre11, Rad50 and Nbs proteins (MRN complex) with C-terminal Binding Protein Interacting Protein (CtIP) is required for MMEJ to remove blockages at the DSB and to initiate end-resection, forming a 3′ overhanging flap [34]. DNA microhomology within the flap allows DNA annealing, with the rest of the flap either being resected or filled in by DNA synthesis activity. More detailed information about MMEJ can be gained from recent reviews [35,36], and both NHEJ and MMEJ repair Cas9 DDSBs in bacteria [37].

**Homologous Recombination** (HR), or homology-directed DNA repair, is a collection of processes in bacteria, archaea and eukaryotes for DDSB repair and to support DNA replication by reactivating replication forks at DNA nicks and barriers [38–41]. HR depends on availability of homologous DNA molecules that can base pair, and in some instances involves strand invasion catalysed by recombinases, RecA, RadA or Rad51 and their accessory and regulatory proteins [42,43]. Two major modes of recombinase-mediated HR operate in many, but not all, bacteria exemplified in *Escherichia coli* by RecBCD and RecFOR pathways [44,45]. RecBCD is a helicase-nuclease that targets dsDNA that is blunt-ended, or recessed by a few nucleotides, and converts them into 3′ ssDNA tailed molecules that are coated by RecA recombinase, through specific interaction between RecBCD and RecA in response to DNA sequences called Chi, reviewed in [45]. The RecFOR complex targets ssDNA gaps that may arise if RecQ helicase unwinds DNA at stalled replication forks or in other contexts such as G4 DNA [46]. RecFOR replaces single-strand DNA binding protein (SSB) with RecA provoking strand invasion into a homologous duplex that can lead to later stages of HR, including Holliday Junction formation by the RuvABC resolvasome.

In human cells, HR is predominant during DNA synthesis (S) and the second growth (G_2_) phases of the cell cycle [47,48]. In HR, DDSBs are recognised by the MRN complex, which recruits CtIP initiating end resection of the DDSB in a 5′–3′ direction, leaving 3′ overhangs. RPA is recruited to 3′ tailed ssDNA that protects DNA and recruits ATR protein. RPA is exchanged with Rad51 recombinase through interaction with BRCA2 and/or RAD52, preparing the 3′ ssDNA tail DNA for strand invasion into homologous DNA template [49,50]. Strand invasion forms a D-loop (‘Displacement-loop’) intermediate, which can be resolved through multiple pathways of double-strand break repair (DSBR), synthesis-dependent strand annealing (SDSA) or break-induced replication (BIR). In these instances recombinase catalysed strand invasion to form a D-loop is a pre-requisite. HR in the guise of single-stranded template repair (SSTR) is similar to SDSA and BIR but does not require a recombinase and therefore does not generate a D-loop [51]. Knowledge of SSTR in cells other than yeasts is very limited but it is a process that is potentially significant for HR-based genome editing that utilises ssDNA as a donor for insertion into a target site.

## Functional interplay of natural CRISPR-Cas and DNA repair in bacteria

Little is known about interactions of DNA repair and Cas9 in *Streptococcus* species, the original source of Cas9, or with Cas12a in its host species of *Acidaminococcus, Francisella* and *Lachnospiraceae*. Most knowledge about how DNA repair and CRISPR-Cas systems interact physically and functionally is currently from the CRISPR-Cas system of *E. coli*, where they have been studied for their effects on CRISPR-Cas ‘Adaptation’, processes that lead to new DNA being integrated into a CRISPR locus by Cas1–Cas2 proteins thus generating or updating immunity*.* The helicase/translocase activities of RecBCD seem to be important for adaptation but the mechanism is not yet known [52–54]. Adaptation is stimulated in *E. coli* by R-loop interference complexes in ‘primed’ or ‘targeted‘ adaptation [55,56], which seems to be a more general effect of interference on adaptation in bacteria [57]. Binding of Cas ribonucleoprotein complexes to target DNA forms R-loops that are sites positively identifying an MGE or other sequence to which the cell has acquired immunity. In this way cross-talk between interference complexes and adaptation reactions can stimulate immunity in response to incursion by an MGE. Genetic analyses in *E. coli* identified that loss of RecG or PriA helicase activities resulted in a loss of primed adaptation, but had no effect on naïve adaptation that occurs without interference R-loops [54]. This, and other genetic data, indicated that RecG and possibly PriA support adaptation through having an effect on R-loop interference complexes. *In vitro*, RecG protein dissociates Cascade interference R-loops that had blocked reconstituted DNA replication [58]. A model was proposed that RecG processes interference R-loops, as part of its intrinsic response to maintaining genome stability, and in doing so generates DNA substrates suitable for capture as new spacers for Adaptation [58]. R-loops trigger genome instability, therefore, mechanisms to dissolve R-loops are widespread across species [59,60]. The principle that RecG helicase can remove Cascade interference R-loops in *E. coli* may be of interest for potential effects of analogous helicases in eukaryotic cells because such enzymes might antagonise genome editing by targeting Cas9 R-loops for removal.

## Interplay of CRISPR-Cas and DNA repair: genome editing

The CRISPR-Cas interference enzyme Cas9 has been used for a variety of gene editing applications in many species, including human cells as reviewed most recently in [61]. The CRISPR-Cas adaptation protein complex Cas1–Cas2 has been used in novel ways to create a CRISPR locus with DNA-based digital witness and recorder properties [62]. We herein focus on Cas9-based editing procedures, for which there is rapidly growing body of information about interplay with DNA repair, and which is likely to be relevant and extended to other genome editing enzymes, most notably Cas12a, as more is understood about their use. Genome editing using Cas9 relies on its natural enzymatic activities, forming an interference R-loop (modified as sgRNA) and generating DDSB, but also activities from Cas9 variants that nick only one DNA strand, lack any nuclease activity or are fused to other enzyme functionalities, the latter described more below.

### NHEJ-based editing: DNA cut, disrupt or re-write

The error-prone nature of NHEJ has been widely used with Cas9-sgRNA for generating gene knockouts, since its inception in 2013 [12]. Two more recent applications of NHEJ, CRISPaint (CRISPR-assisted insertion tagging) and VIKING have been used to re-write DNA sequence information at Cas9-sgRNA targets and are readily available in kit-form. CRISPaint has been used to facilitate tagging of target proteins by editing the target gene with sequence encoding an in-frame ‘tag’ (e.g. luciferase or a coloured fluorescent protein) [63]. VIKING technology was developed from principles of NHEJ that were used in ZFN and Talen-based genome editing [64], for example the ObLiGaRe method [65]. This is modified in VIKING by use of Cas9-sgRNA to direct linearisation of DNA to a VKG1 sequence that is shared widely among plasmid vectors. This optimises binding of the Ku complex and the likelihood that donor DNA will be incorporated into the cut site on the human genome. However, DNA integrations off-target are a concern as is the production of In/Del mutations at DNA junctions surrounding the large inserts and also notes the potential for inserts to be inserted in the reverse conformation, resulting in a failure to express the large cassette which has been inserted [64]. MMEJ is also being exploited for gene editing, benefiting from its activity throughout the cell cycle. MMEJ-based PITCh (Precise Insertion into Target Chromosome) [66,67] has been used to insert custom DNA cassettes flanked by arms of microhomology into a Cas9-induced DDSB with higher efficiency than HR, and mHAX (microhomology assisted excision) [68] is used for scar-less removal of selectable markers from DNA insertions, for example removal of a puromycin cassette from *HPRT1* in human stem cells. Procedures based on MMEJ offer an alternative-editing route to NHEJ and HR, with potentially reduced error rate relative to NHEJ and higher rate of incidence compared with HR.

### HR and Fanconi anaemia pathway based editing: DNA cut and replace

HR can be exploited for ‘genetic replacement’ by insertion of new DNA sequence at the Cas9 R-loop target site. A great deal of effort has been made to optimise HR-based genome editing because it has the ability to accurately swap undesired DNA sequence for a desired sequence, for example to achieve therapeutic editing within mutated cells. The low prevalence of HR throughout the eukaryotic cell cycle and difficulty preparing suitable DNA donor for successful insertion and resistance to cellular assault have stimulated research to optimise genome editing that is underpinned by HR. Strategies for optimisation include: (i) promoting HR over NHEJ in cells, (ii) determining the most suitable combination of genome editing tools for use by insertion into recipient cells, and (iii) determining host cell DNA repair enzymes that promote HR at editing sites, and which antagonise it.

- NHEJ can be suppressed using small molecule inhibitors or gene silencing of genes encoding NHEJ proteins, concomitantly promoting HR-based editing [69–71]. Use of NU7441 and KU-0060648 to inhibit DNA-PKcs in human HEK293 T/17 cells achieved this, with HR measured in a ‘Traffic Light Reporter’ assay as green fluorescence, and NHEJ as Red fluorescence [69]. The study observed a reduction in NHEJ events by 40% and a two-fold increase in successful HR. Use of Scr7, a small molecule inhibitor of the DNA ligase IV DNA binding domain, achieved several-fold increased HR-mediated insertions into various genetic loci in eukaryotic cells [72]. A potential drawback to NHEJ inhibitors however may be that although achieving the intended effect on genome editing it may also lead to problems for DNA repair elsewhere in the genome that requires NHEJ, leading to unintended genome instability away from the Cas9 target site.
- The tools necessary for HR-based genome editing are Cas9 and one or more sgRNAs alongside a donor DNA molecule that contains desired sequence for insertion (Figure 2). One area for optimisation of editing is the composition of donor DNA, which can be linear or circular dsDNA, or a single-stranded donor oligonucleotide (ssODN). Various studies have shown profound differences in editing efficacy depending on donor DNA used, for example [73]. The DNA used comprises DNA arms of sequence homologous to the genome target site that flank the desired DNA sequence. This allows homologous DNA pairing to be initiated after Cas9 has generated a DDSB. Use of phosphothioate-modified oligonucleotides in donor DNA can improve the efficiency of gene modification by stabilising donor DNA against host cell degradation [74]. This is thought to stimulate HR because a higher concentration of template persists for prolonged availability for successful insertion. Similarly, in ssODN higher efficiencies of gene insertion can be achieved compared with dsDNA donor, and can be further improved by chemical modification of ssODN donor [74,75].
- The enhanced effect of ssODN donor DNA on genome editing has placed it at the forefront of establishing how cell DNA repair systems recombine this donor into the chromosome. Genetic replacement using ssODNs is thought to rely on HR repair pathways SDSA and/or SSTR, and the latter is gaining significant new interest because it is many times more active in human cells than recombinase-dependent HR that relies on synapsis forming D-loops [76–79]. Fanconi anaemia (FA) pathway proteins are identified as crucial for genome editing via ssODNs independently of Rad51-mediated HR, and this may rely on localisation of FANCD2 to sites of Cas9-catalysed DDSBs [75]. In this model, FA proteins marshal DNA break repair away from NHEJ and towards SSTR. DNA repair enzymes downstream of FA proteins were also identified as being important for SSTR including Rad51 paralogues, CtIP and HelQ helicase [75]. Knockdowns of HelQ had a strong negative effect on the incidence of SSTR that may be related to its physical interactions with FANCD2 and Rad51 paralogues [75]. HelQ in human cells helps to maintain genome stability by repair of broken down DNA replication and it may act to limit HR from progressing into Holliday junctions [80,81]. The importance of HelQ for this type of genome editing may help to identify more precisely its cellular role.

## Designer Cas9 proteins

Cas9-sgRNA ‘off-the-peg’ catalyses R-loop interference reactions triggering a DDSB at the site of the R-loop. This has also facilitated development of gene editing technologies based on modifying the protein architecture of wild-type Cas9, nuclease inactivated Cas9 (dCas9) and single-strand cutting ‘nickase’ Cas9 (nCas9) including transcriptional regulation and imaging, reviewed recently in [61]. Cas9 protein fusions have also been generated to enhance HR in human cells, by biasing DNA repair pathway choice at the site of the DDSB. Two examples have fused CtIP and RAD52 to Cas9 [82,83].

The CtIP fusion protein was explored using two different methods. An active Cas9-CtIP fusion was able to stimulate an increase in editing compared with standard HR, and a second Cas9-fusion enhanced HR further by fusing an N-terminal fragment of CtIP, deemed the HR-enhancer domain (HE), that is crucial to its initiation of HR [82]. The RAD52 fusion protein was designed with the same rationale of forcing a protein crucial to the completion of HR close to the site of a Cas9 DDSB and was found to enhance the efficiency of reporter cassette insertion [83].

**Figure 3:**
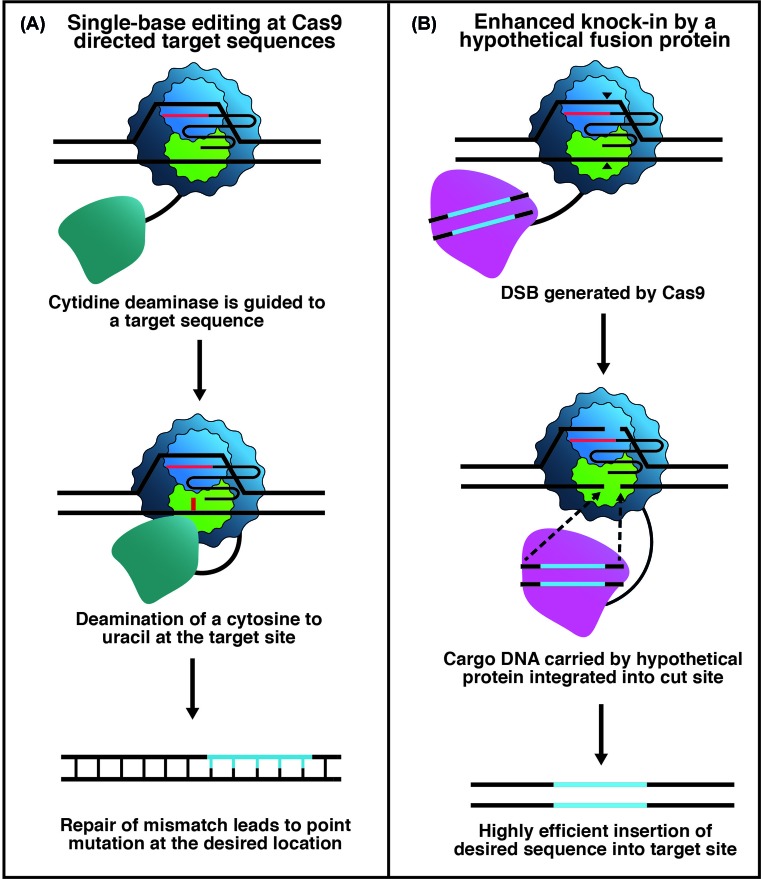
Schematic of genome editing triggered by Cas9-fusions (**A**) A Cas9—CDA fusion protein precisely targets a cytosine base for conversion into uracil. Then, in cahoots with other factors, this can promote C to T transitions in Cas9—targeted DNA sequences. (**B**) Speculative model showing Cas9 fused to a protein for delivering duplex DNA into a site of Cas9 DDSB that may not need to rely on host cell HR processes.

Fusions of dCas9 or nCas9 to DNA base modifying enzymes have facilitated editing of single bases. A cytidine deaminase (CDA)—dCas9 fusion has generated a C to T transition at R-loop targeted cytosine residues by generation of uracil, which is replaced with thymine during subsequent DNA repair (Figure 3) [84]. The R-loop generated window of ssDNA allows the deaminase to convert C into U. By fusing uracil glycosylase inhibitor (UGI) to the C-terminus of CDA—dCas9 base-excision repair was prevented, allowing mismatch repair (MMR) to complete the C to T change [84]. The system was enhanced by fusion of a second UGI, to further favour MMR, and the Gam protein derived from bacteriophage, which binds the free ends of DDSBs, minimising In/Del generation. An adenine deaminase fused to dCas9 has been effective at targeted conversion of adenine into inosine, which is in turn converted into guanine [85]. A similar system, RNA Editing for Programmable A to I Replacement (REPAIR), has also been reported for the single-base editing of adenosine to guanine through an inosine intermediate in RNA transcripts in mammalian cells utilising catalytically inactive Cas13, a class 2 CRISPR-Cas RNA editing enzyme [86]. Finally, the reliance of HR-based CRISPR-Cas9 genome editing on host cell HR enzymes might make it attractive to develop a Cas9 fusion to proteins that are active as site-specific recombinases or have similar DNA integration activity (Figure 3). Cas9-mediated R-loop formation would in this scenario target DNA for integration of a duplex DNA payload carried by the fusion enzyme.

## Concluding remarks

DNA repair was first implicated in cell survival in the 1930s [87], recombination as a form of DNA repair was modelled first in the 1970s [88], but CRISPR-Cas immunity was discovered recently, in 2005–2007. Their combined study is mutually beneficial for improving genome editing towards therapeutic advances in many organisms, but also for understanding human DNA repair processes, which when faulty lead to diseases associated with genome instability, and when activated are obstacles to cancer treatments through helping cancerous cells overcome chemotherapeutic agents.

## Abbreviations

BIR: break-induced replication
Cas: CRISPR-associated
CDA: cytidine deaminase
CRISPaint: CRISPR-assisted insertion tagging
CRISPR: clustered regularly interspaced short palindromic repeat
crRNA: CRISPR-encoded RNA
CtIP: C-terminal binding protein interacting protein
DDSB: DNA double-strand break
D-loop: displacement-loop
FA: Fanconi anaemia
HR: homologous recombination
In/Del: insertion/deletion
MGE: mobile genetic element
MMEJ: microhomology-mediated end joining
MMR: mismatch repair
MRN: complex of Mre11, Rad50 and Nbs protein
NHEJ: non-homologous end joining
RPA: replication protein A
SDSA: synthesis-dependent strand annealing
sgRNA: single-guide RNA
SSTR: single-stranded template repair
ssODN: single-stranded donor oligonucleotide
UGI: uracil glycosylase inhibitor
